# Micronucleated Erythrocytes in Newborn Rats Exposed to Raltegravir Placental Transfer

**DOI:** 10.1155/2014/851820

**Published:** 2014-05-25

**Authors:** Blanca Miriam Torres-Mendoza, Damharis Elizabeth Coronado-Medina, Belinda Claudia Gómez-Meda, Eduardo Vázquez-Valls, Ana Lourdes Zamora-Perez, María de Lourdes Lemus-Varela, Guillermo Moisés Zúñiga-González

**Affiliations:** ^1^División de Neurociencias, Centro de Investigación Biomédica de Occidente, Instituto Mexicano del Seguro Social, 44340 Guadalajara, JAL, Mexico; ^2^Departamento de Clínicas Médicas, Centro Universitario de Ciencias de la Salud, Universidad de Guadalajara, 44350 Guadalajara, JAL, Mexico; ^3^Maestría en Nutrición Clínica, Universidad del Valle de Atemajac, 45050 Zapopan, JAL, Mexico; ^4^Departamento de Biología Molecular y Genómica, Centro Universitario de Ciencias de la Salud, Instituto de Biología Molecular en Medicina y Terapia Génica, Universidad de Guadalajara, 44350 Guadalajara, JAL, Mexico; ^5^Unidad Médica de Alta Especialidad, Hospital de Especialidades, Centro Médico Nacional de Occidente, Instituto Mexicano del Seguro Social, 44340 Guadalajara, JAL, Mexico; ^6^Departamento de Clínicas Odontológicas Integrales, Centro Universitario de Ciencias de la Salud, Instituto de Investigación en Odontología, Universidad de Guadalajara, 44350 Guadalajara, JAL, Mexico; ^7^Departamento de Neonatología, Unidad Médica de Alta Especialidad, Hospital de Pediatría, Centro Médico Nacional de Occidente, Instituto Mexicano del Seguro Social, 44340 Guadalajara, JAL, Mexico; ^8^Laboratorio de Mutagénesis, Centro de Investigación Biomédica de Occidente, Instituto Mexicano del Seguro Social, Sierra Mojada 800, Colonia Independencia, 44340 Guadalajara, JAL, Mexico

## Abstract

The use of raltegravir in treating HIV/AIDS has been proposed due to its effectiveness in suppressing high loads of HIV RNA in pregnant women, thus preventing infection of the fetus. However, administration of raltegravir during pregnancy produces a compound which is transferred to high concentrations to the offspring. The objective of this study is to evaluate the transplacental genotoxic effect of raltegravir in newborn rats. We evaluated the number of micronucleated erythrocytes (MNE), micronucleated polychromatic erythrocytes (MNPCE), and polychromatic erythrocytes (PCE) in the peripheral blood samples of the offspring of Wistar rats treated 6 days before birth with oral administration of raltegravir. The animals were randomly assigned to five groups as follows: raltegravir at doses of 15, 30, or 60 mg/day, cyclophosphamide 10 mg/kg (positive control), or 0.5 ml of sterile water (negative control). In addition, the effect of these drugs on the weight and height of newborns was assessed. There were no differences in the number of MNE, MNPCE, and PCE, and a slight decrease in the weight and height was observed in the offspring of the rat mothers treated with raltegravir. Genotoxicity studies are required in pregnant women to determine the risk of using raltegravir to the fetuses.

## 1. Introduction

Raltegravir or Isentress is the first antiretroviral integrase inhibitor approved by the US Food and Drug Administration in 2009 [1] and the European Medicines Agency in 2007 [2]. Raltegravir prevents the integrase enzyme from incorporating HIV DNA into the host cell, reducing viral replication [3, 4].

Raltegravir is among the available treatments used in highly active antiretroviral therapy (HAART) in treatment-naïve patients with HIV/AIDS as was recommended in current guidelines [5], due to raltegravir being considered as a preferred treatment among the regimens for antiretroviral therapy-naïve patients regardless of the baseline viral load or CD4 cell count, accompanied by a low potential for drug interactions, both in naïve and treated individuals [6].

Raltegravir clearance mechanism is by glucuronidation [7] which reduces its toxicity and protects the fetus from toxic drugs, such as antiretroviral and chemotherapeutic agents [8].

This drug is an alternative for patients with virologic failure to multidrug-resistant HIV [3, 4], with the advantage of not requiring protease inhibitors to potentiate its effect [9].

The use of raltegravir has been proposed because it suppresses high viral loads of HIV RNA in pregnant women in order to prevent the infection of the fetus [10–14].

Raltegravir is a substrate of P-glycoprotein, which is found in high concentrations in the placenta [3, 15], and it is transferred in higher concentrations to offspring (from double to triple raltegravir concentration), apparently due to the low molecular weight and lipophilicity of the raltegravir [11], as well as the reduced ability to bind to proteins (83%) rather than protease inhibitors [15].

Developing medications for humans requires determining the efficiency and effectiveness as well as evaluating the adverse effects, particularly those relating to damage to nucleic acids. This potential genotoxic effect can be measured by the increase in micronucleated erythrocytes (MNE) frequency [16].

The formation of the MNE involves the chromosomes or fragments not integrating into the nucleus of the daughter cells during mitosis and is a good test for assessing the potential genotoxic effects of drugs [17].

No genotoxic and mutagenic effects with raltegravir were discovered by Ames test with* Salmonella typhimurium* and* Escherichia coli* as well as in studies of micronuclei in mice [18]. However, it is necessary to know the genotoxic and cytotoxic potential of transplacental exposure to high doses of raltegravir that could affect the offspring [15].

Reports of European Medicine Agency [2] and FDA [18] show* in vitro* and* in vivo* genotoxicity studies in rat and dog; however, transplacental genotoxicity has not been evaluated in the offspring treated with raltegravir [19].

Therefore, the objective of this study was to determine the transplacental genotoxic effect of raltegravir on newborn rats.

## 2. Material and Methods

### 2.1. Animals

We used the rat transplacental model described by Gómez-Meda et al., [16], which is based on exposing pregnant rats to the test agent from day 16 to 21 of gestation (the final stage of the organogenesis) in order to minimize toxicity to the developing fetuses. We then evaluated whether the agent administered to the mother could cause harmful effects on the fetuses because an increase in MNE was observed in the peripheral blood of neonates. This MNE increase provides information regarding the possible genotoxicity and teratogenic potential of the test agent [16].

Twenty 12-week-old female Wistar rats were mated with males of the same strain. All animals were healthy and were supplied by the laboratory animal facility of the Centro de Investigación Biomédica de Occidente, Instituto Mexicano del Seguro Social (Guadalajara, México), with the approval of the Local Committee on Health Research and the Institutional Committee for the Care and Use of Laboratory Animals (registry number R-2012-1305-4). This study complied with Norma Official Mexicana NOM.062-ZOO-1999, the international institutes of health for the humane treatment of research animals [20], and the ARRIVE guidelines for reporting animal research [21].

The rats were housed in polycarbonate cages in a windowless room with automated temperature control (22 ± 2°C), light control (lights on at 07:00 and off at 19:00 h), and relative humidity maintenance (50 ± 10%). The animals received standard laboratory pelleted food (Purina, México) and tap water* ad libitum*.

### 2.2. Mating of Rats

Pregnancy was determined by vaginal cytology. Each female rat was flushed daily with a vaginal wash of 0.1 mL of water using an adjustable-volume pipettor, and the contents were smeared onto clean slides. The presence of sperm indicated that mating had occurred, and the day sperm was first detected and established at the first day of pregnancy. When pregnancy was confirmed, the female rats were housed in individual cages.

### 2.3. Study Groups and Micronuclei Induction

The rats were distributed at random into five groups of four rats each, and we began a different treatment for each group on the 16th day of gestation.

The dosage of raltegravir (Isentress, Merck & Co., Inc., Whitehouse Station, NJ, USA) was determined based on the human therapeutic dosing (400 mg twice daily, orally), for the average human body weight of 60 kg [22] and multiplying by 10, because it has been described that experimental animals are generally less vulnerable than humans, by a factor of approximately 10 [23]. The groups were as follows: raltegravir 15 mg/day, 30 mg/day, or 60 mg/day, positive control with 10 mg/kg cyclophosphamide (CP) diluted in 0.5 mL of sterile water, and negative control with 0.5 mL sterile water. The treatment was administered orally using an orogastric cannula once daily for 6 days.

### 2.4. Sample Preparation and Micronuclei Analysis

Five pups per dam were randomly selected at birth, and the weight and height of each pup were measured immediately after birth [24]. A drop of peripheral blood was extracted from the tip of the tail of each newborn rat, and two smears were made on precoded slides. All samples were air-dried, fixed in absolute ethanol for 10 min, and stained with acridine orange for analysis [25]. The samples were scored manually and blindly using an Olympus BX51 fluorescence microscope (Olympus, Tokyo, Japan) and an oil-immersion objective (100x). To detect accumulated and recent damage, the number of MNE in 10000 total erythrocytes (TE: polychromatic erythrocytes (PCE) or young cells and normochromatic erythrocytes (NCE) or mature red blood cells) and micronucleated polychromatic erythrocytes (MNPCE) in 1000 PCE were counted. In addition, the number of PCE in 1000 TE was evaluated to determine cytotoxicity as an internal technique control.

### 2.5. Statistical Analysis

The MNPCE, MNE, and PCE frequencies from the five pups per litter were averaged separately to obtain single values for each litter. The results were shown per 1000 erythrocytes (‰) as the mean ± standard deviation (SD).

All the data were tested for normality using the Kolmogorov-Smirnov test. The results were evaluated using the Statistical Program for Social Sciences (version 15.0; SPSS, Inc., Chicago, Illinois, USA) by means of analysis of variance (one-way ANOVA). The Bonferroni test for multiple* post hoc* pair-wise comparisons was employed to correct the significance values for intergroup analysis. A *P* value less than 0.05 was considered statistically significant.

## 3. Results and Discussion

There were no statistically significant differences in the mean frequencies of MNE, MNPCE, and PCE in rat pups from raltegravir-treated mothers compared with the negative control group. However, the positive control group presented elevated MNE and MNPCE frequencies (*P* < 0.0001), whereas the PCE frequencies were reduced (*P* < 0.007) (Table 1). In addition, we analyzed the differences among neonate rat weights and heights at birth, and these parameters indicated a decrease (*P* < 0.0001) in the three raltegravir groups compared with the negative control group (Table 2).

There are approximately 25 drugs approved for clinical use in patients with HIV/AIDS worldwide that may cause side effects and/or fetal toxicity via maternal exposure for antiretroviral therapy [26–28].

This study used a transplacental model, which allows for evaluating the genotoxic susceptibility to drugs by the fetus through the increase in MNE frequency because their immunologic immaturity mainly demonstrated the ability to clearance by macrophages in the spleen and metabolic hypofunction [29–32]. The genotoxic evaluation of antiretroviral drugs, including raltegravir, might be considered due to the high frequency of cancer in patients with AIDS [33].

As expected, in this study, compared with the negative control, the MNE and MNPCE frequencies increased in the positive control group due to the micronucleogenic effect of this compound. In addition, the PCE frequency values decreased significantly due to the cytotoxic effect of cyclophosphamide. However, the raltegravir-treated groups did not exhibit genotoxic or cytotoxic effects, but common side effects as diarrhea were observed in these rats.

Antiretrovirals for HIV/AIDS are classified according to their mechanism of action for HIV/AIDS in six groups. The genotoxic activity of antiretrovirals has been demonstrated using reverse transcriptase inhibitors thymidine analogs, such as 3′-azido-3′-deoxythymidine (AZT, zidovudine) alone or in combination with dideoxyinosine (ddI), and is capable of altering chromosomal integrity in newborns in murine models, which have potentially adverse effects similar to those discovered in humans [34, 35]. For example, AZT combined with 5-fluorouracil used in colon cancer showed genotoxic damage [36].

Raltegravir belongs to a family of integrase inhibitors whose safety in pregnant women has not been established [37]. Raltegravir acts as integrase strand transfer inhibitor to treat HIV.

Although pregnant women receiving raltegravir transferred high concentrations of the compound to their newborns [15], in this study, the absence of MNE supports the hypothesis that raltegravir does not participate in DNA fragmentation in the host cell [38].

The activity of UGT-dependent pathways to clearance raltegravir in the fetus or neonate is reduced, perhaps due to the immaturity or reduced transcription, however, is increased in pregnant women [15]; apparently it also avoids the genotoxic effect.

This absence of genotoxic effects suggests that the administration of raltegravir in pregnant women does not require an adjustment of the drug. However, raltegravir is not a substrate of cytochrome P450 enzymes, which may contribute to the absence of interaction and reduce adverse effects with drugs metabolized by this pathway, but it is not expected to affect the pharmacological action of these drugs [39].

In relation to the characteristics of weight and height, the pups transplacentally exposed to cyclophosphamide in this study displayed differences in weight and height compared with the negative control group because of the cytotoxic effect of cyclophosphamide. There were also changes in weight and height in the raltegravir groups.

Consistent with the above, the presence of HIV infection in children slightly slows growth [40]. In addition, 12%–48% of women infected with HIV have newborns with a low birth weight [41], and the use of antiretroviral drugs in pregnant HIV mothers decreased the frequency of low birth weight [42].

Overall, HAART improves the anthropometric measures of children [43], and antenatal HAART-exposed versus non-HAART-exposed uninfected children demonstrated minimal differences in weight and height [40]. However, high doses of raltegravir in animal models, such as rats and dogs, caused low birth weights [2], similar to the results observed in this study.

Case report studies have justified the use of raltegravir to reduce high viral loads in pregnant women [11, 14]; however, it has been found that raltegravir administered did not reduce the weight in the newborn [14], while, controversially, Croci et al. have reported low birth weight of newborns of mothers treated with raltegravir [3].

Although pups born from raltegravir-treated rats demonstrated a reduction in height and weight in this study, this effect could be due to common side effects in rats such as diarrhea. However, transplacental effect of raltegravir on the weight and height should be evaluated in future studies.

## 4. **Conclusions**

In conclusion, the results suggest that raltegravir administration to pregnant rats could induce a reduction in weight and height in the offspring of mothers treated during pregnancy; however, no genotoxicity effects in offspring were demonstrated by the transplacental micronuclei test. Raltegravir could be considered to evaluate its effectiveness in pregnant women with HIV/AIDS principally in the last trimester of pregnancy and eventually could be recommended especially in pregnant women who have high viral loads or who are drug resistant. However, additional human studies will be necessary to further improve our understanding of individual susceptibility to this treatment.

## Figures and Tables

**Table 1:** MNE, MNPCE, and PCE frequencies (‰) of the samples analyzed.

Study groups	MNE	MNPCE	PCE
Negative control: sterile water	1.6 ± 0.6	3.8 ± 1.6	358.3 ± 59.6
Dose 1: raltegravir 15 mg/day	1.5 ± 0.4 NS	3.1 ± 1.5 NS	397.6 ± 50.7 NS
Dose 2: raltegravir 30 mg/day	1.6 ± 0.5 NS	3.6 ± 1.9 NS	360.0 ± 66.3 NS
Dose 3: raltegravir 60 mg/day	1.7 ± 0.5 NS	4.2 ± 1.8 NS	354.7 ± 80.0 NS
Positive control: cyclophosphamide 10 mg/kg/day	48.6 ± 16.3 *P* < 0.0001	92.3 ± 47.4 *P* < 0.0001	287.4 ± 71.3 *P* = 0.007

Data (‰) are expressed as mean ± SD; *n* = 5/group; NS: not significant; MNE: micronucleated erythrocytes/1000 TE; TE: total erythrocytes; MNPCE: micronucleated polychromatic erythrocytes/1000 PCE; PCE: polychromatic erythrocytes/1000 TE. All groups were compared versus the negative control.

**Table 2:** Weight and height in newborn rats from mothers treated during pregnancy.

Study groups	Weight (g)	Height (cm)
Negative control: sterile water	6.5 ± 0.3	5.0 ± 0.2
Dose 1: raltegravir 15 mg/day	5.9 ± 0.4 *P* < 0.0001	4.8 ± 0.2 *P* = 0.001
Dose 2: raltegravir 30 mg/day	6.1 ± 0.5 *P* < 0.0001	4.8 ± 0.3 *P* = 0.009
Dose 3: raltegravir 60 mg/day	6.3 ± 0.5 NS	4.8 ± 0.4 *P* = 0.002
Positive control: cyclophosphamide 10 mg/kg/day	4.3 ± 0.2 *P* < 0.0001	4.4 ± 0.2 *P* < 0.0001

Data are expressed as mean ± SD; *n* = 5/group; NS: not significant. All groups were compared versus the negative control.
